# Perioperative Factors and Radiographic Brixia Scores’ Effect on Early Extubation After Fallot Tetralogy Surgery

**DOI:** 10.3390/jcm15041409

**Published:** 2026-02-11

**Authors:** İbrahim Akkoç, Selin Sağlam, Ezgi Direnç Yücel, Hatice Dilek Özcanoğlu, Erkut Öztürk, Ali Can Hatemi, Funda Gumus Ozcan

**Affiliations:** 1 Department of Anaesthesiology and Reanimation, Saglik Bilimleri University, Basaksehir Cam and Sakura Hospital, Istanbul 34480, Turkey; selin.saglam2@saglik.gov.tr (S.S.); ezgidirenc.yucel@saglik.gov.tr (E.D.Y.); dilekmersin@saglik.gov.tr (H.D.Ö.); funda.gumusozcan@sbu.edu.tr (F.G.O.); 2 Department of Pediatric Cardiology, Saglik Bilimleri University, Basaksehir Cam and Sakura Hospital, Istanbul 34480, Turkey; erkut.ozturk@saglik.gov.tr; 3 Department of Pediatric Cardiovascular Surgery, Saglik Bilimleri University, Basaksehir Cam and Sakura Hospital, Istanbul 34480, Turkey; alican.hatemi@sbu.edu.tr

**Keywords:** children, Fallot tetralogy, early extubation

## Abstract

**Introduction and Objective:** This study aims to evaluate the effect of perioperative factors and radiographic Brixia scores on early extubation following corrective surgery for Fallot tetralogy at a high-volume single cardiac center. **Materials and Methods:** A retrospective evaluation was conducted on 120 cases who underwent complete correction due to Fallot tetralogy [Median age 6 months (IQR 5–7), Median weight 6.2 kg (IQR 5.2–8 kg)]. Patient demographics, preoperative characteristics, intraoperative variables, postoperative outcomes, surgical type, surgical duration, cardiopulmonary bypass (CPB) time, cross-clamp time, and blood product volumes were retrieved from electronic medical records. P/F ratio, PaO_2_/FiO_2_, and Oxygen Index (OI) were calculated. Early extubation was defined as extubation occurring within 6 h after the completion of surgery. The Brixia score (Interstitial opacities, 1 point; interstitial predominant alveolar, 2 points; and interstitial and alveolar opacities, 3 points) was graded for both lung lobes divided into three segments, with a total score ranging from 0 to 18. The results were analyzed statistically. **Results:** In 60% of the cases (*n* = 72), valve-preserving surgery was performed, and in 40% (*n* = 48), a transannular patch was used. The early extubation rate was 20% (*n* = 24). The median duration of mechanical ventilation was 10 h (IQR, 6–15). Older age (median 8 vs. 5 months), valve-preserving surgery, lower incidence of right-to-left shunt Patent Foramen Ovale (63% vs. 84%), higher P/F ratio on ICU admission (360 vs. 220), and lower Brixia scores on ICU admission (8 vs. 11) and on postoperative day 1 (7 vs. 12) were identified as significant factors for early extubation (*p* < 0.05). The mortality rate in the entire patient group was 3.3%. In multivariable logistic regression analysis, older age (OR: 1.2, 95% CI: 1.1–1.9 *p* = 0.03), valve-sparing repair (OR: 1.7, 95% CI: 1.2–2.5, *p* = 0.008), and lower postoperative Brixia scores (OR:1.4 95% CI: 1.2–2.1, *p* = 0.02) remained independently associated with early extubation. **Conclusions:** The Brixia score can be used as a reliable scoring system for evaluating postoperative lung status. Pulmonary valve-preserving repair shows a profile of earlier lung parenchyma recovery compared to transannular patch repair.

## 1. Introduction

Fallot tetralogy (TOF) is the most common cyanotic congenital heart disease (CHD) that requires surgical intervention, constituting 7–10% of all congenital heart diseases [1]. In TOF surgery, different techniques for complete correction can be used, including transannular patch repair; pulmonary valve-preserving repair; or in some cases, repair without right ventriculotomy [2,3]. TOF consists of four classical anatomical features: ventricular septal defect, right ventricular outflow tract obstruction, overriding aorta, and right ventricular hypertrophy. These structural abnormalities result in reduced pulmonary blood flow and chronic hypoxemia, which can manifest clinically as cyanosis, exercise intolerance, feeding difficulty, and delayed growth in infancy. If untreated, progressive RV hypertrophy and hypoxia may lead to arrhythmia, cerebral abscesses, and thromboembolic complications in later life. Therefore, early surgical correction is essential for improving long-term survival and functional capacity in these patients.

In recent years, improvements in TOF surgery and postoperative intensive care have led to better outcomes in terms of mortality and morbidity. Despite a decrease in the age at surgery, published reports show that mortality rates range between 1–4%. However, risks such as hemodynamically significant residual defects and prolonged intensive care unit (ICU) stays still remain [2,3]. Historically, surgical management of TOF evolved from palliative shunt procedures in the 1950s to modern complete repair strategies performed during infancy. The shift towards early primary repair has been driven by improvements in cardiopulmonary bypass, myocardial protection, and perioperative care. However, the choice between transannular patch (TAP) repair and valve-sparing repair remains a topic of debate, as TAP provides wider outflow but carries a risk of lifelong pulmonary regurgitation, whereas valve-preserving surgery aims to maintain valve competence and potentially reduce long-term right ventricular dilation.

Early extubation following pediatric heart surgery has become increasingly common. The primary goal of this approach is to avoid the adverse effects of positive pressure ventilation on the cardiovascular system, reduce airway irritation, and prevent complications such as ventilator-associated pneumonia [4]. Specifically, after TOF surgery, early extubation has been reported to provide hemodynamic benefits by increasing blood pressure and cerebral oxygen saturation [5]. However, due to the negative effects of cardiopulmonary bypass (CPB) and sudden hemodynamic changes, the optimal timing and location for extubation remain controversial. The concept of fast-track extubation is based on minimizing exposure to mechanical ventilation, promoting spontaneous breathing, and enhancing cardiac output by reducing intrathoracic pressure. Nevertheless, pediatric patients differ from adults in terms of airway anatomy, lung compliance, and inflammatory response to CPB, making the decision for extubation more delicate. Factors such as age, weight, syndromic status, surgical complexity, and perioperative hemodynamics play a key role in extubation timing. Identifying objective radiological or physiological markers that can guide this decision may improve postoperative outcomes and shorten ICU stay.

While PaO_2_/FiO_2_ (P/F) ratio and Oxygen Index (OI) are commonly used to assess oxygenation effectiveness in patients, these measurements may not accurately reflect pulmonary function in the presence of significant intracardiac shunts. A few studies have suggested that a simple radiographic scoring system, such as the Brixia score, could be helpful in evaluating pulmonary status [6]. Brixia scoring, originally developed for COVID-19 related lung involvement, quantifies alveolar and interstitial opacities by assigning segmental scores ranging from 0 to 18. Its simplicity, reproducibility, and accessibility without advanced imaging make it attractive in pediatric cardiac ICUs, where daily chest radiographs are routinely obtained. Unlike isolated gas exchange parameters, Brixia reflects structural lung pathology, potentially offering earlier detection of atelectasis, pulmonary edema, or ventilation-perfusion mismatch. Therefore, it may serve as a decision-support tool for predicting extubation readiness after TOF repair.

Given the limited evidence integrating radiographic scoring systems with perioperative clinical variables in TOF surgery, further investigation is needed to determine whether Brixia scoring can support early extubation strategies. We hypothesized that lower Brixia scores, together with favorable perioperative parameters, would be associated with shorter ventilation duration. In this study, we evaluated perioperative factors and postoperative Brixia scores to identify predictors of early extubation following complete TOF repair.

## 2. Materials and Methods

This study was conducted retrospectively on pediatric patients under 18 years of age who underwent complete corrective surgery for Fallot Tetralogy (TOF) between 1 August 2020, and 1 December 2024. Patients with known neurological diseases, those who had undergone shunt surgery prior to the study, those who died within the first 24 h, and those who had previously been dependent on mechanical ventilation were excluded from the study. The study was designed in accordance with the tenets of the Declaration of Helsinki and obtained the necessary approval from the local ethics committee (Protocol Code: 2025/394).

A study form was created for each case, containing the patient’s demographic information, preoperative characteristics, intraoperative variables, postoperative outcomes, type of surgery, surgical duration, cardiopulmonary bypass (CPB) time, cross-clamp time, and volumes of blood products used. The cases were divided into two groups: the early extubation group, consisting of patients extubated within the first 6 h after the operation in the intensive care unit (ICU), and the delayed extubation group, consisting of patients extubated after the 6th hour of their ICU stay following the operation [7].

Extubation criteria were defined as: staying awake without any stimulus, presence of spontaneous respiratory effort, positive end-expiratory pressure (PEEP) of 5 cm H_2_O, partial pressure of carbon dioxide (PaCO_2_) ≤ 50 mmHg, pH ≥ 7.25, stable hemodynamic status, absence of systolic and diastolic dysfunction on echocardiography, and a good cough reflex and swallowing function. Reconnection of an extubated patient to a mechanical ventilator within 1 h was defined as reintubation [8].

All cases were planned for extubation as soon as possible. In perioperative anesthesia management, appropriate medications were administered following the protocol used in the clinic where this study was conducted. According to the protocol, for children aged six months, 0.1 mg/kg midazolam, 1 μg/kg fentanyl, and 0.6 mg/kg rocuronium were administered at induction; and for maintenance, 0.1 μg/kg/minute remifentanil, 5 μg/kg/minute rocuronium, and a minimum alveolar concentration of 1–1.2 sevoflurane were used. For children older than six months, 0.1 mg/kg midazolam, 1 μg/kg fentanyl, and 0.6 mg/kg rocuronium were given at induction; and for maintenance, 0.25 μg/kg/minute remifentanil, 5 μg/kg/minute rocuronium, a minimum alveolar concentration of 1–1.2 sevoflurane, and 0.5 μg/kg/hour dexmedetomidine were administered. The effects of neuromuscular agents were antagonized by sugammadex. Remifentanil and rocuronium were discontinued after the sternum was closed, sevoflurane was discontinued before the skin was closed, and dexmedetomidine was continued until the patient was transferred to the pediatric cardiac intensive care unit [7]. Patients were started on 0.5 μg/kg/minute milrinone at the beginning of the operation. The maintenance dose was determined by the anesthetist on a case-by-case basis, taking into account the initiation of catecholamine and the patient’s hemodynamic status during volume management.

The P/F ratio was calculated as PaO_2_/FiO_2_. Oxygen Index (OI) was calculated as mean airway pressure × FiO_2_ ×100/PaO_2_ [6]. The Brixia score, as previously described, was adopted to measure lung abnormality. In this scoring system, the lungs were divided into six regions, and the degree of opacity was scored according to its location as follows: Each zone is scoring as: Score 0 no lung abnormalities, Score 1 interstitial infiltrates, Score 2 interstitial and alveolar infiltrates (interstitial predominance), Score 3 interstitial and alveolar infiltrates (alveolar predominance). The total of the scores for the six lung regions was reported as the Brixia score for each radiograph (minimum 0, maximum 18) [9]. The Brixia score was calculated using chest radiographs obtained preoperatively (one day before surgery), immediately after surgery (Brixia PCICU), and on postoperative day 1 (POD 1).

Postoperative transthoracic echocardiography was performed routinely in all patients within 24 h of the surgery, usually before extubation in the intensive care unit. Right-to-left or left-to-right shunting through a patent foramen ovale (PFO) was detected by postoperative transthoracic echocardiography using color Doppler imaging

Valve-preserving surgery was defined as a transatrial and transpulmonary approach aimed at avoiding a ventricular incision and preserving a competent pulmonary valve. The valve-preserving approach for Tetralogy of Fallot was generally applied in infants without severe annular hypoplasia (z-score ≥ −3).

## 3. Statistical Analysis

The distribution of variables was analyzed using computer software. Descriptive values were obtained using the SPSS (Version 25.0) (Statistical Package for the Social Sciences for Windows) software package and expressed as median [interquartile range (IQR)] and percentage-percentile values. Chi-squared test and Mann–Whitney U test were used to compare the variables between groups. Multivariable logistic regression analysis was performed to identify independent predictors of early extubation. The model was adjusted for clinically relevant covariates, including age, sex, body weight, cardiopulmonary bypass time, aortic cross-clamp time, valve-sparing repair, and postoperative Brixia score. The results are reported as odds ratio (OR) with 95% confidence intervals (CI). A *p*-value < 0.05 was considered statistically significant. In addition to group comparisons, postoperative parameters were evaluated at multiple time points (ICU admission, 6th hour, postoperative day 1) to observe temporal changes in oxygenation indexes and Brixia scores. Correlations between Brixia score, P/F ratio and Oxygen Index were examined to assess the relationship between radiographic lung involvement and oxygenation efficiency. Where appropriate, Fisher’s exact test was applied for categorical variables with low expected frequency. Continuous data that did not meet the assumption of normal distribution were compared using non-parametric tests.

## 4. Results

A total of 120 patients who underwent complete repair for Tetralogy of Fallot were included in the study. Early extubation was achieved in 24 patients (20%), while 96 patients (80%) were extubated after 6 h. Demographic, perioperative and postoperative variables of the two groups were compared in Table 1.

Patients in the early extubation group had significantly higher age and weight compared with the delayed extubation group [Age: 8 (6–10) vs. 5 (4–6) months, *p* < 0.001; Weight: 8 (6–10) vs. 5.5 (5–6) kg, *p* < 0.001]. Mechanical ventilation duration was markedly shorter in the early group [4 (3–5) vs. 14 (10–18) hours, *p* = 0.005], parallel to reduced PCICU stay [56 (48–64) vs. 80 (72–96) hours, *p* = 0.01] and total hospitalization days [7 (6–8) vs. 10 (8–12), *p* = 0.02].

There was no significant difference in CPB time or packed red blood cell requirement between groups. Oxygenation parameters revealed that the P/F ratio at PCICU admission was notably higher in the early extubation group [360 (320–400) vs. 220 (190–260), *p* < 0.001], while Oxygen Index values were lower [OI: 4 (3–5) vs. 6 (4–7), *p* < 0.001]. Although P/F and OI values tended to remain better at 6 h and day 1 in early extubation patients, the difference for day-1 P/F was not statistically significant.

Radiographic assessment demonstrated a strong association between postoperative lung involvement and extubation outcome. Brixia scores were significantly lower in the early extubation cohort both on PCICU admission [8 (5–10) vs. 11 (9–14), *p* < 0.001] and on postoperative day 1 [7 (6–9) vs. 12 (10–15), *p* < 0.001], indicating a more favorable lung status. Valve-sparing repair was also more common in early-extubated patients (84% vs. 54%, *p* = 0.010), while PFO persistence was more frequent in delayed extubation group (84% vs. 63%, *p* = 0.020). Reintubation and mortality rates did not differ significantly between groups.

In multivariable logistic regression analysis, older age (OR: 1.2, 95% CI: 1.1–1.9 *p* = 0.03), valve-sparing repair (OR: 1.7, 95% CI: 1.2–2.5, *p* = 0.008), and lower postoperative Brixia scores (OR:1.4 95% CI: 1.2–2.1, *p* = 0.02) remained independently associated with early extubation.

## 5. Discussion

In this study, the effect of perioperative factors and radiographic Brixia scores on extubation before 6 h after complete correction surgery for Fallot tetralogy (TOF) was evaluated. Older age, valve-sparing surgery, lower incidence of right-to-left shunt PFO, higher P/F ratio on ICU admission (360 vs. 220), lower Brixia scores on ICU admission (8 vs. 11), and on postoperative day 1 (7 vs. 12) were found to be significant factors for early extubation. Our study is one of the limited studies where such evaluations were conducted.

The reported early extubation rates in the literature range widely from 27% to 82% [7,10,11]. The primary reason for this variation is the use of different time intervals to define early extubation. Some authors consider extubation performed in the operating room as early extubation, while others consider extubation within the first hour, the first 6 h, the first 12 h, or the first 24 h after surgery. In our study, we considered extubation within the first 6 h as early extubation. In this case, our early extubation rate was 20%.

Numerous preoperative, intraoperative, and postoperative factors have been reported to affect early extubation. One study reported that older age, higher body weight at the time of surgery, shorter CPB time, and the use of ultrafiltration were independent risk factors associated with early extubation [8]. In another recent study, factors such as age, body weight, the presence of a syndrome, surgical risk category, and operation time were found to influence extubation time [7]. In our study, older age and weight, along with valve-sparing repair, were associated with early extubation in cases undergoing TOF surgery.

The primary reason for avoiding early extubation in congenital heart surgery is the frequent need for reintubation. In a multicenter pediatric cardiac intensive care study conducted after neonatal cardiac surgery, the reported reintubation rate was 11% [12]. Tirotta et al. [13] reported reintubation rates of 2.9% in the early extubation group, 3.9% in the delayed extubation group, and 3.4% across the entire cohort in a study of 637 infant cases. In our study, the reintubation rates were 4.4% in the early extubation group, 3.1% in the delayed extubation group, and 3.3% across all cases.

Postoperative pulmonary complications are more frequent in pediatric patients undergoing cardiac surgery with cardiopulmonary bypass (CPB), with a reported risk of up to 25% in different studies [14]. Factors such as decreased chest wall compliance, mucus plugs, small airways, weakened cough reflex, low birth weight, plastic bronchitis, pulmonary hypertension, genetic abnormalities, ventilator-associated pneumonia, and atelectasis may worsen pulmonary complications [14,15]. PaO_2_/FiO_2_ (P/F), Oxygen Index (OI), and arterial blood gas parameters are commonly used, but they may be misleading in the presence of intracardiac shunts [6]. Chest X-rays, although unable to detect lung involvement in the early stages of the disease, can be useful as a fast and effective diagnostic tool for monitoring critically ill patients in the intensive care unit. Chest X-rays can reflect lung abnormalities and disease severity [16]. To standardize this, various scoring systems have been developed, and Brixia is one of the scoring systems increasingly used in recent years. Brixia scoring of chest X-rays has been shown to predict the severity and mortality of COVID-19 [16]. Additionally, it has been reported that it can predict the effectiveness of oxygen therapy and shows a high correlation with OI [17,18]. In a study evaluating TOF cases, early extubation was considered as extubation within 12 h. They found that the Brixia score on the 12th hour (7 vs. 12) and on the first postoperative day (9 vs. 12) was significantly lower in the early extubation group [6]. In our study, similar results were observed. We found that the Brixia score was superior to P/F and OI in predicting early extubation. This result may have been influenced by the presence of PFO.

The ideal surgery for TOF is complete correction that minimizes the need for re-interventions. The recommended age for complete correction is between 3–6 months for asymptomatic cases. Early repair with a transannular patch used to be the preferred approach for providing an unrestricted RVOT with free pulmonary insufficiency. These cases are at risk for low cardiac output and restrictive right ventricle development in the early period [19]. Monocusp valve implantation has been attempted to improve cardiac output by reducing PR when functionally adequate, but the results have been inconsistent, likely due to variations in the techniques and materials used [20]. Valve-sparing repair (VSR) is gaining popularity, and there is still a balance between choosing TAP and VSR among surgeons. A recent study evaluated 259 cases of TOF surgery, and VSR was preferred in 62% of the cases [6]. In our unit, the surgical approach is based on balancing annular adequacy, valve quality, and the ability to create an efficient outflow tract with <2/3 systemic right ventricular pressures. Valve-sparing surgery was performed in 60% of the cases.

## 6. Limitations

The main limitations of this study are that it is a single-center, retrospective analysis, which may limit the generalizability of the results to other institutions with different clinical practices or postoperative protocols. The sample size, although adequate for preliminary evaluation, may not be sufficient to perform extensive multivariable modeling or determine strong predictive cut-off values for Brixia scoring in early extubation. Another limitation is the lack of comprehensive assessment of postoperative complications such as atelectasis severity, ventilator-associated infections, pulmonary hypertension episodes, and long-term respiratory outcomes, which could provide additional insight into lung recovery profiles. Surgical techniques were categorized as valve-preserving or transannular patch repair, but subgroup comparisons related to patch size, monocusp use, and annular z-scores could not be evaluated in detail. Additionally, follow-up imaging beyond postoperative day 1 was not included, which may have underestimated the progression or resolution of radiographic findings over time. Prospective, multicenter studies with larger cohorts and extended postoperative radiologic follow-up are needed to validate these findings and establish a more objective use of Brixia scoring in clinical decision-making.

## 7. Conclusions

In conclusion, the Brixia score appears to be a useful, simple and reproducible radiographic tool for evaluating postoperative lung status after TOF repair. Lower Brixia scores were associated with higher P/F ratios and more favorable early extubation outcomes, suggesting that radiographic lung assessment may aid clinicians in predicting readiness for fast-track recovery protocols. Pulmonary valve-sparing repair demonstrated a trend toward quicker pulmonary recovery and shorter ventilation duration compared to transannular patch procedures, supporting its preferential use when anatomically feasible. Integrating radiographic scoring with perioperative hemodynamic and oxygenation parameters may help refine extubation strategies, reduce ICU stay and improve patient recovery. Larger prospective trials with longer follow-up are required to confirm the predictive value of Brixia scoring and to determine its role within standardized postoperative extubation algorithms.

## Figures and Tables

**Table 1:** Comparison of Patients According to Early and Late Extubation.

	Early Extubation (*n* = 24)	Delayed Extubation (*n* = 96)	*p*
Age	8 (6–10)	5 (4–6)	<0.001
Weight, kg	8 (6–10)	5.5 (5–6)	<0.001
Sex			NS
Male	14 (58)	50 (52)	
Female	10 (42)	46 (48)	
Mechanical ventilation, hour	4 (3–5)	14 (10–18)	0.005
Pediatric Cardiac intensive care unit, hour	56 (48–64)	80 (72–96)	0.01
Hospital, day	7 (6–8)	10 (8–12)	0.02
Cardiopulmonary bypass, minutes	120 (110–130)	130 (115–145)	NS
Packed red blood cells	2 (1–3)	2 (1–3)	NS
P/F PCICU admission	360 (320–400)	220 (190–260)	<0.001
P/F PCICU 6 h	300 (260–330)	250 (220–280)	NS
P/F Day 1	270 (250–290)	250 (210–270)	NS
OI PCICU admission	4 (3–5)	6 (4–7)	<0.001
OI PCICU 6 h	2.5 (2–3)	3 (2–4)	0.02
OI Day 1	2 (1.5–2.5)	2.5 (2–3)	NS
Brixia one day before surgery	5 (4–8)	5 (3–8)	NS
Brixia PCICU	8 (5–10)	11 (9–14)	<0.001
Brixia Day 1	7 (6–9)	12 (10–15)	<0.001
Preop RVOT gradient(mmHg)	60 (50–70)	65 (50–75)	NS
Postop RVOT gradient(mmHg)	15 (10–20)	10 (5–15)	NS
Patent foramen ovale Post-repair	15 (63)	81 (84)	0.020
Valve sparing repair	20 (84)	52 (54)	0.010
Transannular patch repair	4 (16)	44 (46)	
Reintubation	1 (4.4)	3 (3.1)	NS
Mortality	-	4 (3.3)	NS

*n* (%) or Median (Interquartile range), NS: non-significant; OI: Oxygenation index; PCICU: Pediatric Cardiac Intensive care unit; RVOT: Right ventricle outlet tract.

## Data Availability

The datasets generated and analyzed during the current study are not publicly available due to patient confidentiality but are available from the corresponding author upon reasonable request.
